# Early use of renin–angiotensin–aldosterone system inhibitors and stable renal function in familial focal segmental glomerulosclerosis with *ACTN4* mutation: a case report and literature review

**DOI:** 10.1186/s12882-025-04441-4

**Published:** 2025-09-26

**Authors:** Kisho Miyasako, Yujiro Maeoka, Yuho Adachi, Ryo Tamura, Naoki Ishiuchi, China Nagano, Kandai Nozu, Takao Masaki

**Affiliations:** 1 Department of Nephrology, Hiroshima University Hospital, 1-2-3 Kasumi, Minami-ku, Hiroshima, Hiroshima 734-8551 Japan; 2 Department of Pediatrics, Kobe University Graduate School of Medicine, 7-5-1 Kusunoki-cho, Chuo-ku, Kobe, Hyogo 650-0017 Japan

**Keywords:** Alpha-actinin-4, Nephrotic syndrome, Steroid-resistant nephrotic syndrome, Focal segmental glomerulosclerosis, Angiotensin-converting enzyme inhibitor, Inherited kidney disease, Cyclin D1

## Abstract

**Background:**

Genetic mutations in alpha-actinin 4 (*ACTN4*) are one cause of familial focal segmental glomerulosclerosis (FSGS) and steroid-resistant nephrotic syndrome (SRNS) in early adulthood, eventually progressing to end-stage kidney disease. Early initiation of renin–angiotensin–aldosterone system inhibitors (RAASis) is reported to delay progression of several forms of familial FSGS and SRNS; however, no cases involving *ACTN4* mutations have been reported.

**Case presentation:**

A 16-year-old boy was admitted to our hospital for a detailed evaluation of proteinuria that first appeared during treatment for Duchenne muscular dystrophy (DMD) and persisted for 1 year. He had been treated with prednisolone and an angiotensin-converting enzyme inhibitor (ACEi) for 2 years prior to the onset of persistent proteinuria. A renal biopsy revealed segmental sclerosis in 1 of 40 glomeruli, with effaced foot processes observed under electron microscopy. Genetic testing identified *ACTN4* mutation (c·776C > T, p.T259I), leading to a diagnosis of autosomal dominant FSGS caused by *ACTN4* mutation. After the first appearance of proteinuria, the patient’s renal function and urinary protein levels remained stable for following 7 years.

**Conclusions:**

We report a case of adolescent-onset FSGS with *ACTN4* mutation diagnosed during ACEi therapy for the prevention of DMD-associated cardiomyopathy. The patient exhibited stable renal function and no disease progression compared with his father and previously reported cases with the same variant. This is the first reported case of early RAASi induction for treating *ACTN4*-associated FSGS with long-term stable renal function.

## Background

Primary nephrotic syndrome is a glomerular disease characterized by severe proteinuria and a major cause of end-stage kidney disease (ESKD) in both children and adults [1]. Approximately 5–20% of children and up to 40% of adults with primary nephrotic syndrome are resistant to steroid therapy, resulting in progression to ESKD [2–5]. This condition is known as steroid-resistant nephrotic syndrome (SRNS); it is the second most common cause of ESKD within a patient’s first 20 years of life [6] and results in a poor prognosis. The predominant renal histopathology of SRNS is frequently focal segmental glomerulosclerosis (FSGS), a pathology influenced by factors including genetic mutations, infections, immune disorders, drugs, renal hemodynamics, and obesity [7–10]. While most cases of FSGS arise sporadically with no family history, recent advances in next-generation sequencing and human genome databases have rapidly accelerated the identification of monogenic variants linked to SRNS [7, 11]. A recent study reported that genetic FSGS accounts for up to 30–60% of SRNS cases in infants and young children [12]. Additionally, 25–30% of SRNS in patients under 25 years old are associated with single-gene mutations [6, 13], suggesting that familial FSGS is more prevalent than previously recognized. From almost 50 genes identified as causative factors of FSGS and/or SRNS, mutations in *ACTN4*, which encodes alpha-actinin-4, account for approximately 2% of cases, and *ACTN4* is the third most common gene implicated in autosomal dominant FSGS in Europe and the United States, following *INF2* (9–17%) and *TRPC6* (3%) [14–17]. ACTN4 stabilizes the actin cytoskeleton of foot cells by cross-linking fibrous actin in a lattice-like lateral direction in the kidneys [18–20]. Therefore, patients with *ACTN4* mutations develop an autosomal dominant form of FSGS, presenting with subnephrotic proteinuria, foot process effacement, and initial proteinuria or nephrotic syndrome in early adulthood, which often progresses to renal failure without ocular, neurologic, or myopathic deficits [9, 21, 22].

No established therapy exists for most forms of familial FSGS and SRNS. Although glucocorticoids and calcineurin inhibitors are often used empirically, these immunosuppressive agents are frequently ineffective in treating these conditions. Renin–angiotensin–aldosterone system inhibitors (RAASis), including angiotensin-converting enzyme inhibitors (ACEis) and angiotensin II receptor blockers, are fundamental therapeutic agents used to slow chronic kidney disease progression [23]. RAASis exert their renoprotective effects through mechanisms involving lowering intraglomerular pressure by decreasing efferent arteriolar pressure, restoring the size and charge selectivity to the glomerular cell wall, and reducing production of transforming growth factor-beta, a key driver of glomerulosclerosis and fibrosis [24, 25]. Therefore, RAASis are also used as supportive therapy for nephrotic syndromes, including FSGS, and chronic glomerulonephritis such as IgA nephropathy [26, 27]. However, few case studies have reported the efficacy of RAASi for treating familial FSGS and SRNS [28–31]. Notably, in patients with Alport syndrome—a genetic glomerular disease that can progress to SRNS—early ACEi initiation significantly delayed disease progression, particularly in those with microalbuminuria [32]. Similarly, few cases have demonstrated the benefits of early RAASi initiation in treating familial FSGS [31], and no cases involving ACTN4 mutations have been reported to date.

Here, we report a case of adolescent-onset FSGS with *ACTN4* mutation (c.776C > T, p.T259I), diagnosed by genetic testing following a renal biopsy performed for persistent proteinuria observed during treatment for Duchenne muscular dystrophy (DMD). The patient had been receiving ACEi therapy for 2 years prior to the onset of persistent proteinuria. The patient had a family history of ESKD, and based on this, his father underwent genetic testing and was found to carry the same *ACTN4* variant. Although his father’s medical records were unavailable because the retention period had expired at the treating hospital, his father developed proteinuria at a similar age and eventually progressed to hemodialysis within 10 years despite glucocorticoid treatment. Compared with his father and previously reported cases with the same variant, the patient maintained with no disease progression over 7 years. While direct comparison is limited due to the unknown penetrance of this variant and possible intra familial variability related to environmental and lifestyle factors, the patient’s stable renal function raises hypothesis that initiating RAASi therapy during the period of mild proteinuria may delay FSGS progression.

## Case presentation

A 16-year-old Japanese boy with no chief complaints was admitted to our hospital for a thorough examination of his proteinuria that had persisted for more than 1 year. At the age of 6, he was diagnosed with DMD at our hospital. A few years before admission, he began taking prednisolone to treat his DMD, and alfacalcidol, famotidine, enalapril, and carvedilol to mitigate the adverse effects of prednisolone and to prevent DMD-associated cardiomyopathy. Significantly, the initiation of these medications was prior to the onset of proteinuria. Although his father had renal dysfunction in high school and underwent a renal biopsy, the cause of the renal dysfunction was undetermined, resulting in his undergoing hemodialysis at the age of 26. The patient’s mother was a carrier of DMD, and two of his maternal cousins developed DMD.

On admission, he was 156.0 cm tall (−2.44 standard deviation [SD]), weighed 69.1 kg (+0.87 SD), had a BMI of 28.3, and had central obesity owing to long-term prednisolone use. His blood pressure was normal (119/66 mmHg), and no edema was observed. The initial laboratory evaluation (Table 1) showed a normal complete blood count; serum creatinine, 0.33 mg/dL; estimated glomerular filtration rate using creatinine (eGFR_Cr_): 294.4 mL/min per 1.73 m^2^; serum cystatin C: 0.94 mg/dL; eGFR cystatin C (eGFR_Cys_): 95.9 mL/min per 1.73 m^2^; total protein 7.2 g/dL: albumin 4.5 g/dL. His aspartate aminotransferase, alanine aminotransferase, lactate dehydrogenase, and creatine kinase were elevated owing to DMD before the appearance of proteinuria, with no change from baseline values. Urinalysis revealed his proteinuria was 2+ and urine–protein–creatinine ratio (uPCR) was 1.05 g/gCr. Serologic workup results were mostly negative. Ultrasonography of the kidneys showed that they were of normal size.

**Table 1:** Patient’s laboratory characteristics on admission

Parameter	Value	(normal range)
(Urine)		
pH	6.0	
Urine protein/creatinine ratio (g/gCr)	1.05	( < 0.15)
Urine protein by 24-hour urine storage (g/day)	0.54	( < 0.15)
Red blood cell (/HPF)	0	( < 5)
Oval fat body	Positive	Negative
(Blood)		
White blood cell (/μL)	5560	(3040–8540)
Neutrophil (%)	52.0	(38.3–71.1)
Eosinophil (%)	4.0	(0.2–7.3)
Basophil (%)	2.0	(0.2–2.0)
Lymphocyte (%)	37.0	(21.3–50.3)
Monocyte (%)	4.0	(2.7–7.6)
Red blood cell (10^4^/μL)	523	(378–499)
Hemoglobin (g/dL)	15.3	(10.8–14.9)
Hematocrit (%)	45.9	(35.6–45.4)
Platelet (10^4^/μL)	26.4	(15.0–36.0)
AST(U/L)	144	(13–33)
ALT(U/L)	175	(8–42)
Creatine kinase (U/L)	10128	(59–248)
Total cholesterol (mg/dL)	147	(142–248)
Triglyceride (mg/dL)	131	(40–234)
Low-density lipoprotein cholesterol (mg/dL)	82	(70–139)
High-density lipoprotein cholesterol (mg/dL)	39	(38–90)
Choline-esterase (U/L)	316	(240–486)
Total protein (g/dL)	7.2	(6.7–8.3)
Serum albumin (g/dL)	4.5	(4.0–5.0)
Blood urea nitrogen (mg/dL)	9.4	(8–20)
Creatinine (mg/dL)	0.33	(0.40–0.70)
eGFR_Cr_ (mL/min/1.73 m^2^)	294.4	( > 90)
Cystatin C (mg/L)	0.94	(0.63–0.94)
eGFR_Cys_ (mL/min/1.73 m^2^)	95.9	( > 90)
Na (mmol/L)	140	(138–146)
K (mmol/L)	4.3	(3.6–4.9)
Cl (mmol/L)	104	(99–109)
Calcium (mg/dL)	10.2	(8.6–10.4)
Phosphate (mg/dL)	3.9	(2.5–4.7)
Uric acid (mg/dL)	6.8	(2.3–7.0)
Plasma glucose (mg/dL)	121	(70–109)
Hemoglobin A1c (NGSP) (%)	5.6	(4.6–6.2)
C-reactive protein (mg/dL)	0.28	( < 0.20)
IgG (mg/dL)	969	(870–1700)
IgA (mg/dL)	156	(110–410)
IgM (mg/dL)	96	(46–260)
CH50 (IU/mL)	50.2	(30–46)
C3 (mg/dL)	123	(86–160)
C4 (mg/dL)	29	(17–45)
Anti-nuclear antigen	Negative	Negative
HBs-Ag	Negative	Negative
HCV-Ab	Negative	Negative

HPF, high-power field; AST, aspartate transaminase; ALT, alanine transaminase; eGFR, estimated glomerular filtration rate; Cr, creatinine; Cys, cystatin C; IgG, immunoglobulin G; IgA, immunoglobulin A; IgM, immunoglobulin M; HBs-Ag, hepatitis B surface antigen; HCV-Ab, hepatitis C antibody

**Fig. 1:**
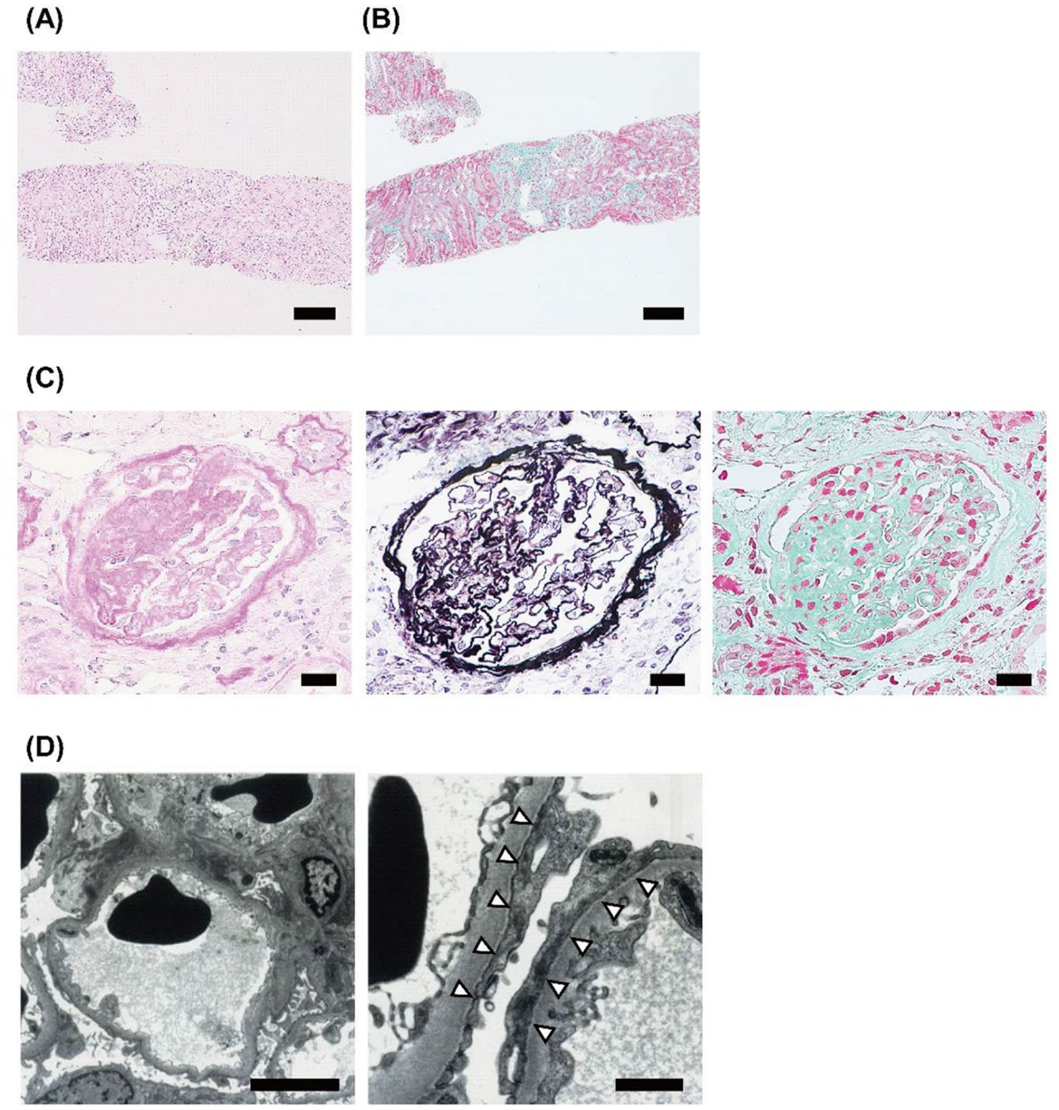
Light and electron microscopic findings on renal biopsy (**A**) Hematoxylin–eosin (HE) staining and (**B**) Masson’s trichrome (MT) staining at × 100 magnification on the renal biopsy. Minimal fibrosis of the renal cortex was observed. Scale bars: 200 μm. (**C**) Segmental sclerotic glomerular images from periodic acid-schiff staining (*left*), co-staining with HE staining and periodic acid-methenamine-silver (*center*), and MT staining (*right*) at × 400 magnification. Segmental sclerosis was observed in 1 of 40 glomeruli. Scale bars: 20 μm. (**D**) Electron microscopic image of the segmentally effaced foot process at × 4000 (*left*, scale bar: 5 μm) and × 15,000 (*right*, scale bar: 1 μm) magnification. Arrowheads indicate areas of effaced foot processes is observed. No mitochondrial abnormalities or electron-dense deposits were observed

A renal biopsy was performed, which yielded 40 glomeruli, including no global sclerosis and one segmental sclerosis (Figs. 1A–1D). The remaining glomeruli were almost normal, with no significant deposition under immunofluorescence (Fig. 1E). Foot processes were segmentally effaced under electron microscopy, with no mitochondrial abnormalities or electron dense deposits detected (Fig. 1F). Based on these findings, the patient was diagnosed with FSGS (not otherwise specified variant). The patient had persistent proteinuria despite treatment with 35 mg/day prednisolone and 10 mg/day enalapril as treatment for DMD and prevention of its complications until the time of the renal biopsy, suggesting that minimal change nephrotic syndrome was clinically unlikely. Given the patient’s young age of onset, paternal history of renal dysfunction, and renal biopsy findings consistent with FSGS, genetic testing was performed after receiving informed consent from the patient and his father. The analysis identified a heterozygous missense mutation, c.776C > T (p.T259I), in exon 8 of *ACTN4* in both the patient and his father, leading to a definitive diagnosis of autosomal dominant FSGS. At the time of genetic diagnosis, the patient exhibited no severe renal dysfunction or proteinuria in the nephrotic range. Therefore, conservative treatment with 35 mg/day prednisolone and 10 mg/day enalapril, which the patient was already taking, was continued. The latest laboratory results showed serum cystatin C 0.89 mg/dL and uPCR: 0.21 g/gCr, with routine outpatient follow-up after discharge from the hospital. No progression of renal dysfunction or increase in proteinuria has occurred over the 7 years to date.

## Discussion and conclusions

We report the case of a patient with a family history of ESKD who developed adolescent-onset familial FSGS caused by *ACTN4* mutation. Although the c.776C > T (p.T259I) mutation of *ACTN4* has been previously identified (Table 2), detailed clinical information remains limited. This case contributes to the literature by documenting a long-term clinical course with stable renal function, in contrast to the rapid progression described in some previously reported cases with *ACTN4* mutations.

**Table 2:** List of cases reported to date on *ACTN4* mutations

Patient number (Ethinicity)	*ACTN4***mutation**	Gender	Age at initial symptom	Family history	Symptoms	Proteinuria at biopsy	Origin of varient	Histopathologic diagnosis (Type of variant)	Treatment	Time to ESKD	Age of kidney transplantation (Time to reintroduction)	Pathogenicity	Refference
1 (Chinese)	c.1–34C > T (None)	N/A	N/A	Sporadic	N/A	N/A (non-nephrotic range)	N/A	FSGS (N/A)	N/A	N/A	N/A	Likely pathogenic	[33]
2 (South African)	c.174C > T (p.A58A)	N/A	N/A	N/A	NS	N/A	N/A	N/A	N/A	N/A	N/A	Probably not	[34]
3 (Western European)	c.175C > T (p.W59R)	M	5 y	FH	Proteinuria (2.46 g/gCr), bilateral high-frequency hearing loss	N/A	*de novo*	FSGS (N/A)	N/A	3 y (Hemodialysis)	10 y (N/A)	Pathogenic	[14]
4 (Chinese)	c.184T > A (p.S62T)	N/A	N/A	Sporadic	N/A	N/A (non-nephrotic range)	*de novo*	FSGS (N/A)	N/A	N/A	N/A	Pathogenic	[33]
5 (White)	c.214 G > C (p.E72Q)	N/A	N/A	N/A	N/A	N/A	N/A	N/A	N/A	N/A	N/A	Pathogenic	[16]
6 (Colombian)	c.445_447del (p.I149del)	N/A	N/A	FH	Proteinuria	N/A	N/A	FSGS (N/A)	N/A	N/A (Typically within 10 y of initial diagnosis)	N/A	Pathogenic	[14]
7 (White)	c.457T > C (p.F153L)	N/A	N/A	N/A	N/A	N/A	N/A	N/A	N/A	N/A	N/A	Likely pathogenic	[16]
8 (N/A)	c.457T > C (p.F153L)	N/A	17 y	N/A	SRNS	15 g/day	N/A	FSGS (N/A)	Cyclosporine A	N/A	N/A	Likely pathogenic	[35]
9 (Caucasian)	c.464T > C (p.I155T)	F	3 y, 8 m	Sporadic	SRNS	N/A	*de novo*	FSGS (N/A)	N/A	N/A	N/A	Pathogenic	[36]
10 (Czech)	c.475T > C (p.S159P)	F	15 y, 1 m	N/A	SRNS	N/A	N/A	N/A	Cyclosporine A	N/A	N/A	Likely pathogenic	[37]
11 (Chinese)	c.494C > T (p.A165V)	F	17 y	Sporadic	Proteinuria	10.85 g/L/day	*de novo*	N/A	Hemodialysis	Already at the first visit (Hemodialysis)	17 y (N/A)	Pathogenic	[38]
12 (German)	c.584 G > A (p.G195D)	F	13 y	Sporadic	ESKD	2.3 g/m^2^/day	*de novo*	N/A	N/A	Already at the first visit (Peritoneal dialysis)	14 y (N/A)	Pathogenic	[39]
13 (N/A)	c.584 G > A (p.G195D)	N/A	13 y	N/A	ESKD	N/A	*de novo*	FSGS (N/A)	Peritoneal dialysis	Already at the first visit (Peritoneal dialysis)	14 y (N/A)	Pathogenic	[35]
14 (Japanese)	c.671T > C (p.L224P)	M	6 y	N/A	Proteinuria	N/A	*de novo*	FSGS (N/A)	N/A	7 y	N/A	Pathogenic	[40]
15 (N/A)	c.703T > C (p.S262P)	F	15 y	FH	Proteinuria	6.5 g/gCr	N/A	FSGS (N/A)	Glucocrticoid	3 y	20 y (1 y, 6 m)	Pathogenic	[41]
16 (N/A)	c.703T > C (p.S262P)	F	16 y	FH	Proteinuria	N/A	N/A	FSGS (N/A)	N/A	16 y	Performed immediately after dialysis (N/A)	Pathogenic	[41]
17 (N/A)	c.703T > C (p.S262P)	F	20 y	FH	Proteinuria at pregnancy checkups	8.97 g/gCr	N/A	FSGS (N/A)	N/A	3 y	24 y (N/A)	Pathogenic	[41]
18 (N/A)	c.719T > C (p.M240T)	N/A	4 y	N/A	Proteinuria	N/A	N/A	FSGS (N/A)	Cyclosporine A, ACEi (ramipril)	5y, 6 m (PEKT)	9 y (N/A)	Likely pathogenic	[35]
19 (Oklahoma)	c.763A > G (p.K255E)	N/A	N/A	N/A	N/A	N/A	N/A	FSGS (N/A)	N/A	N/A	N/A	Pathogenic	[42]
20 (California)	c.776C > T (p.T259I)	N/A	N/A	N/A	N/A	N/A	N/A	FSGS (N/A)	N/A	N/A	N/A	Pathogenic	[42]
21 * **(Japanese)**	**c.776C > T (p.T259I)**	**M**	**15 y**	**FH**	**Proteinuria**	**1.05 g/gCr**	**N/A**	**FSGS (NOS variant)**	**Glucocrticoid, ACEi (enalapril)**	**N/A**	**N/A**	**Pathogenic**	**Our case (son)**
**22**^†^ **(Japanese)**	**c.776C > T (p.T259I)**	**M**	**16 y**	**FH**	**Proteinuria**	**N/A**	**N/A**	**N/A**	**Glucocrticoid**	**10 y** **(Hemodialysis)**	**28 y** **(18 y)**	**Pathogenic**	**Our case (his father)**
23 (Canary Islands)	c.776C > T (p.T259I)	N/A	N/A	N/A	N/A	N/A	N/A	FSGS (N/A)	N/A	N/A	N/A	Pathogenic	[42]
24 (N/A)	c.776C > A (p.T259N)	M	6 y	N/A	SRNS	N/A	N/A	FSGS (N/A)	N/A	N/A	N/A	Likely pathogenic	[43]
25 (White)	c.778_786del (p.Y260_S262del)	F	12 y, 3 m	Sporadic	SRNS	N/A	N/A	FSGS (Collapsing variant)	N/A	1 y, 4 m	N/A (Performed)	Likely pathogenic	[13]
26 (Caucasian)	c.782C > A (p.V261E)	F	7 y, 7 m	Sporadic	SRNS	N/A	*de novo*	FSGS (N/A)	N/A	8 y, 9 m (N/A)	11 y (N/A)	Pathogenic	[36]
27 (Caucasian)	c.784T > C (p.S262P)	F	5 y	Sporadic	SRNS, facial puffiness	N/A	*de novo*	FSGS (Collapsing variant)	Glucocorticoid, tacrolimus, Rituximab, and ARB (losartan)	6 m (Peritoneal dialysis)	6 y (N/A)	Pathogenic	[44]
28 (Korean)	c.785C > T (p.S262F)	F	3 y	Sporadic	Proteinuria	9.6 g/day	Germline mosaicism from the father’s sperm	FSGS (Collapsing variant)	N/A	N/A	N/A	Likely pathogenic	[45]
29 (Korean)	c.785C > T (p.S262F)	M	3 y, 7 m	Sporadic	Proteinuria	N/A	Germline mosaicism from the father’s sperm	FSGS (N/A)	Glucocorticoid, cyclosporine A, cyclophosphamide, and mycophenolate	N/A	5 y, 7 m (Died on postoperative day 9)	Likely pathogenic	[45]
30 (Europian American)	c.793T > C (p.Y265H)	F	14 y	Sporadic	NS	11.69 g/m^2^/day	N/A	FSGS (Collapsing variant)	Glucocorticoid, ACEi (enalapril) or ARB (losartan), beta blocker, and CCB	6 m (Peritoneal dialysis)	16 y (N/A)	Pathogenic	[46]
31 (Japanese)	c.912 + 1 G > A	M	8 y	N/A	Proteinuria	N/A	N/A	FSGS (N/A)	N/A	N/A	N/A	Likely pathogenic	[40]
32 (White, Black, and Western European)	c.929 G > A (p.R310Q)	N/A	N/A	N/A	N/A	N/A	N/A	FSGS (N/A)	N/A	N/A	N/A	Probably not	[14]
33 (Black)	c.1046A > G (p.Q348R)	F	20 y	Sporadic	Proteinuria	N/A	N/A	FSGS (N/A)	N/A	N/A	N/A	Probably not	[14]
34 (Caucasian)	c.1149C > G (p.I383M)	M	21 y	FH	Proteinuria	N/A	N/A	MCD	N/A	8 y (N/A)	N/A	Probably not	[47]
35 (Czech)	c.1279 G > A (p.A427T)	F	8 m	N/A	SRNS	N/A	N/A	FSGS (N/A)	Cyclosporine A	7 y, 6 m (Peritoneal dialysis)	N/A	Pathogenic	[37]
36 (Czech)	c.1279 G > A (p.A427T)	F	39 y	Sporadic	NS	N/A	N/A	FSGS (N/A)	N/A	N/A	N/A	Pathogenic	[48]
37 (Kurd)	c.1606C > A (p.R536S)	M	N/A	N/A	Proteinuria, hematuria	N/A	N/A	FSGS (N/A)	N/A	N/A	N/A	Likely pathogenic	[49]
38 (Chinese)	c.1649A > G (p.D550G), c.2315C > T (p.A772V)	M	8 y	Sporadic	NS	N/A	N/A	FSGS (N/A)	N/A	N/A	N/A	Likely pathogenic	[50]
39 (Chinese)	c.1649A > G (p.D550G), c.2315C > T (p.A772V)	F	11 y	Sporadic	NS	N/A	N/A	FSGS (N/A)	N/A	N/A	N/A	Likely pathogenic	[50]
40 (Chinese)	c.2191-G > A	M	9 y	Sporadic	NS	N/A	N/A	FSGS (N/A)	Glucocrticoid	N/A	N/A	Likely pathogenic	[50]
41 (Chinese)	c.2191-G > A	M	13 y	Sporadic	NS	N/A	N/A	FSGS (N/A)	Glucocrticoid	N/A	N/A	Likely pathogenic	[50]
42 (Czech)	c.2242A > G (p.N748D)	F	54 y	FH	NS	N/A	N/A	FSGS (N/A)	N/A	N/A	N/A	Pathogenic	[48]
43 (Western European)	c.2401 G > A (p.V801M)	N/A	N/A	FH	N/A	N/A	N/A	FSGS (N/A)	N/A	N/A	N/A	Probably not	[14]
44 (N/A)	c.2511 G > A (p.R837Q)	N/A	N/A	N/A	N/A	N/A	N/A	N/A	N/A	N/A	N/A	Probably not	[14]

This table was created based on data from previous reports [13, 14, 16, 33–50]. This case and the family’s case are shown in bold. N/A, not available; FSGS, focal segmental glomerulosclerosis; NS, nephrotic syndrome; FH, family history; SRNS, steroid-resistant nephrotic syndrome; ESKD, end-stage kidney disease; ACEi, angiotensin-converting enzyme inhibitor: PEKT, preemptive kidney transplantation; NOS, not otherwise specified variant; ARB, angiotensin II receptor blocker; CCB, calcium channel blocker; MCD, minimal change disease. * Our patient and ^†^ his father’s case

Of note, the patient’s father was diagnosed with renal dysfunction and proteinuria at almost the same age and began hemodialysis 10 years later (Table 2), whereas the patient’s renal function and proteinuria remained unchanged after 7 years after the first appearance of proteinuria. The patient was diagnosed with DMD of maternal origin at the age of 6 years. Glucocorticoids and ACEis were initiated a few years before admission to treat his DMD and prevent DMD-associated cardiomyopathy prior to the onset of persistent proteinuria. RAASis are a therapy for various proteinuric diseases in adults and children because of their antiproteinuric and renoprotective effects; they also show efficacy in controlling proteinuria in patients with idiopathic SRNS [9]. RAASis are also reported to be effective in treating genetic FSGS [28–31] and Alport syndrome [32, 51, 52], although evidence for this is limited. Fitzwater et al. demonstrated that monotherapy with RAASis was associated with maintained renal function and markedly reduced proteinuria in 10 pediatric patients with FSGS [30]. Copelovitch et al. reported partial or complete remission of proteinuria achieved by early RAASi induction in three children from two unrelated families with familial FSGS [31]. Similarly, Soliman et al. described the case of a 9-month-old infant with steroid-resistant FSGS who achieved complete remission with RAASis alone [29]. Additionally, RAASi monotherapy reported to be superior to immunotherapy in a cohort of children with familial FSGS in Philadelphia, PA, USA (with 55% Black patients) [28]. Although the lack of detailed clinical information on our patient’s father and the potential for intrafamilial phenotypic variability limit making direct comparisons, the absence of strong evidence supporting glucocorticoid efficacy in familial FSGS supports the possibility that early RAASi introduction may have influenced the differences in clinical progression between our patient and his father. In reported cases of *ACTN4* mutations, RAASis were typically initiated only in patients who already had severe proteinuria or severe renal dysfunction, leading to ESKD within 5 years (Table 2). To our knowledge, no prior reports exist of RAASis being introduced during the period of mild proteinuria (Table 2). Although *ACTN4*-related FSGS is highly, but not fully, penetrant [42], which may account for variability in clinical presentation, early RAASi initiation may be a potential therapy in patients with genetic or familial FSGS caused by *ACTN4* mutations, similar to other genetic FSGS cases. Further studies and additional cases are needed to confirm this approach.

Patients with FSGS findings on renal biopsy should first be evaluated for potential secondary causes requiring specific treatment, such as human immunodeficiency virus-1 infection (HIV) and coenzyme Q10 biosynthesis disruption [7, 9], although the patient in our case had a negative HIV test and no mitochondrial abnormalities in his podocytes. In contrast to genetic FSGS, patients with primary FSGS are initially treated with empirical glucocorticoid therapy [26, 53, 54]. If the disease is resistant to glucocorticoids or refractory or the patient cannot tolerate the adverse effects of the drug, calcineurin inhibitors, alkylating agents, and mofetil mycophenolate are commonly used as the main therapeutic agents in children, and calcineurin inhibitors and mofetil mycophenolate are primarily used in adults [9, 26, 54]. In addition to these immunosuppressive therapies, statins, RAASis, and dietary sodium restriction are common initial treatments for both adults and children [26]. Although some cases of genetic FSGS may respond to calcineurin inhibitors, evidence supporting glucocorticoid therapy for genetic FSGS is insufficient, and such treatment is often ineffective [10, 55]. In the current case, the patient was treated with glucocorticoids and RAASis, but no other immunosuppressive agents. Nevertheless, renal function and urinary protein levels showed favorable trends. These findings suggest that early initiation of RAASis to treat genetic FSGS may help prevent the need for immunosuppressive agents.

The mutation site in this case is located in the actin binding domain (ABD; residues 47–270) of *ACTN4*, which is reported to be associated with a common human renal disease known as familial FSGS [14] (Table 2). The mechanism by which *ACTN4* mutations cause FSGS is not yet fully understood, but several studies have examined the effects of *ACTN4* mutations on protein function in vitro and in vivo. Alfa-actinins are 100-kDa rod-shaped proteins that form head-to-tail homodimers, cross-linking actin filaments and regulating cytoskeletal organization as well as linking the actin cytoskeleton to multiple signaling and membrane proteins [56–60]. Four human alfa-actinin (ACTN1–4) family isoforms encode closely related actin cross-linking proteins [42, 56]. ACTN1 is ubiquitously expressed and most commonly associated with focal adhesions [56, 61, 62]. ACTN2 and ACTN3 are highly expressed in muscle cells and are involved in cross-linking of actin filaments in the Z-disc [63, 64]. ACTN4 is widely expressed and plays a crucial role in maintaining cell shape and structure [61, 62]. In the kidney, ACTN4 is highly expressed in podocytes, binds actin filaments, translocates to the nucleus, and is involved in the transcriptional activity of nuclear receptor [65–69]. In mouse models, ABD mutations in *Actn4* disrupt the gene’s normal localization in actin stress fibers and adhesion plaques and promote formation of actin aggregates in lung fibroblasts and kidney podocytes [18, 70]. The mutation also increases the F-actin binding affinity of Actn4, leading to the formation of abnormal cell aggregates with F-actin in the cells [70, 71]. *Actn4* knockout mice exhibit altered podocyte morphology and develop glomerular disease, eventually leading to renal failure [72]. These mechanisms may contribute to dysfunction of Actn4 and development of FSGS. The c.776C > T (p.T259I) missense mutation in *ACTN4* in our patient was first identified by *Kaplan et al*. in 2000 [42]. Although the detailed clinical course of patients with this variant remains unreported to date, it may cause SRNS in childhood and require renal replacement therapy in adulthood due to progressive renal dysfunction, suggesting that the phenotype is not milder than other *ACTN4* variants (Table 2). This case suggests that early RAASi initiation may be beneficial, potentially independent of the *ACTN4* mutation site, although further studies are needed.

**Fig. 2:**
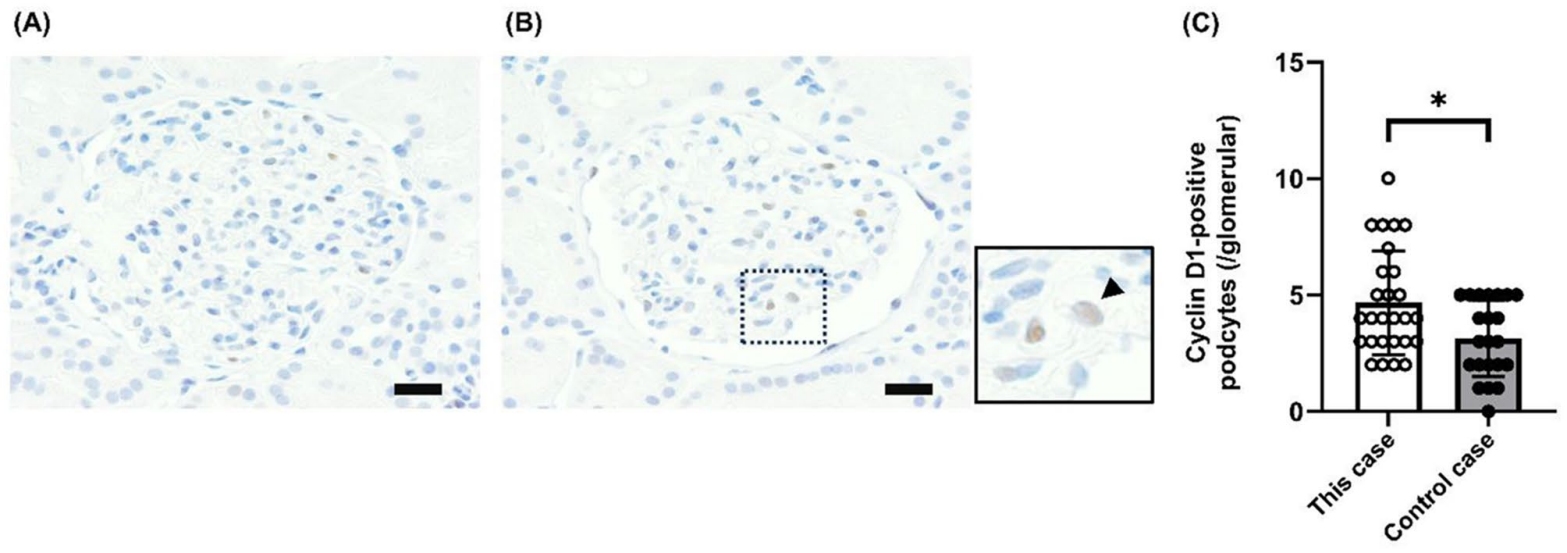
Cyclin D1 staining in patient with *ACTN4* mutation (**A**, **B**) Representative immunohistochemistry staining for cyclin D1 at × 400 magnification revealed several cyclin D1-positive podocytes. Cyclin D1 staining of extracapillary cells was negative in the control patient with minor glomerular abnormalities (**A**) but positive in our patient (**B**). Arrowhead indicates cyclin D1-positive podocyte. Scale bars: 20 μm. (**C**) Quantification graph representing the number of cyclin D1-positive podocytes in both patients. The cyclin D1-positive podocytes were counted and compared for all non-deficient complete glomeruli in each patient. Anti-cyclin D1 (E3P5S) rabbit monoclonal antibody (#55506S, cell signaling technology, Danvers, MA, USA) was used for the primary antibody. Data are presented as means ± standard deviation. ^*^p < 0.05, analyzed by Student’s t-test

In the present case, 1 of 40 glomeruli showed segmental sclerosis, which were unable to make the diagnosis considering family history. However, in early-phase FSGS, diagnosis can be challenging owing to the limited number of glomeruli showing segmental sclerosis, thus a reliable marker to help diagnose FSGS would be highly valuable. Given that glomerular epithelial proliferation is involved in the pathogenesis of FSGS and that cyclin D1–positive podocytes have been reported in an *Actn4*-deficient mouse model of FSGS [73], we performed cyclin D1 staining in this study. The results showed that cyclin D1-positive podocytes significantly increased in our patient compared with that of a control patient with minor glomerular abnormalities (Figs. 2A–2C). Cyclin D1 plays important roles in cell migration, proliferation, differentiation, survival, and apoptosis [73, 74]. It forms a complex with its catalytic partner, cyclin-dependent kinase (CDK4) 4 or CDK6, to drive the cell cycle and cell proliferation/division by regulating progression through the G1 phase and the G1-to-S phase transition, initiating DNA replication [75–77]. The expression, activation, intracellular distribution, stabilization, and degradation of D-type cyclins in normal cells are strictly controlled in response to on/off mitogenic signaling [77, 78]. Conversely, the overexpression, accumulation, and mislocalization of D-type cyclins have been implicated in tumor development and chemotherapy resistance [77, 78]. In renal glomeruli, cyclin D1 expression is weakly positive in the nuclei of endothelial cells and intracapillary cells such as mesangial cells, but is almost negative in the nuclei of extracapillary cells, including podocytes [79]. *Actn4*-knockout mice exhibit an altered podocyte phenotype, with increased numbers of cyclin D1-positive podocytes, which are cell proliferation markers [80]. However, no published reports have described cyclin D1 staining in human renal tissue with *ACTN4* mutations. Interestingly, the number of cyclin D1-positive podocytes was increased even in our patient despite his relatively mild FSGS with proteinuria below the nephrotic range. Although these results are exploratory, are not correlated with disease severity or progression, and require validation in additional patients, these findings offer the possibility that cyclin D1 staining may help in early diagnosis of familial FSGS patients with *ACTN4* mutations, regardless of RAASi administration.

In conclusion, we have diagnosed a case of concomitant FSGS secondary to an *ACTN4* variant (c.776C > T, p.T259I) in a patient with DMD. In this case, RAASi treatment was initiated before the onset of proteinuria, and renal function remained stable over a 7-year course. Considering that early induction of RAASis can delay disease progression in Alport syndrome, and RAASis have been reported to be effective in some FSGS cases [28–32, 51, 52], the favorable clinical course in this case may reflect a beneficial effect of early RAASi initiation, although the possibility of intrafamilial variability may have been present. Therefore, RAASi initiation after diagnosis may be beneficial in patients with familial FSGS caused by *ACTN4*, even if no proteinuria is apparent; thus, more studies are required (similar to Alport disease). This case also provides exploratory evidence that the number of cyclin D1-positive podocytes may be increased in familial FSGS associated with *ACTN4* mutations, highlighting the need to further explore cyclin D1 staining as a potential tool for early diagnosis. To validate these suggestions, further studies are needed in patients presenting early-phase FSGS with *ACTN4* mutation.

## Data Availability

No datasets were generated or analysed during the current study.

## Abbreviations

ESKD: End-Stage Kidney Disease
SRNS: Steroid-Resistant Nephrotic Syndrome
FSGS: Focal Segmental Glomerulosclerosis
ACTN4: Alpha-Actinin-4
RAASi: Renin–Angiotensin–Aldosterone System inhibitor
ACEi: Angiotensin-Converting Enzyme inhibitor
DMD: Duchenne Muscular Dystrophy
eGFR_Cr_: Estimated Glomerular Filtration Rate Using Creatinine
eGFR_Cys_: Estimated Glomerular Filtration Rate Using Cystatin C
uPCR: Urine Protein/Creatinine Ratio
HIV: Human Immunodeficiency Virus-1
ABD: Actin- Binding Domain
CDK: Cyclin-Dependent Kinase
